# Time Alone – Solitude and Loneliness from the Self-Determining Perspective

**DOI:** 10.1007/s12124-026-10039-8

**Published:** 2026-11-03

**Authors:** Radek Heissler, Nikola Doubková, Veronika Juríčková, Karolína Knížková, Marek Preiss, Pavel Mohr

**Affiliations:** 1 National Institute of Mental Health, Klecany, Czech Republic; 2 AMBIS University, Prague, Czech Republic; 3 Department of Psychiatry, First Faculty of Medicine, Charles University and General University Hospital in Prague, Prague, Czech Republic; 4 University of New York in Prague, Prague, Czech Republic; 5 Third Faculty of Medicine, Charles University in Prague, Prague, Czech Republic

**Keywords:** Solitude, Loneliness, Self-determination, Time alone

## Abstract

Solitude and loneliness represent two contrasting yet connected dimensions of being alone, shaped less by physical isolation and more by the motivations underlying it. While loneliness is perceived negatively, linked to distress and adverse outcomes, solitude can be a positive source of self-reflection, personal growth, or relaxation. Drawing on Self-Determination Theory, this article examines how the satisfaction and frustration of basic psychological needs for autonomy, competence, and relatedness influence individuals’ experiences of time alone. We argue that solitude is best understood as a self-determined, intrinsically motivated experience in which the individual voluntarily chooses to be alone in ways that support autonomy, competence, or relatedness needs. In contrast, loneliness is conceptualized as a non-self-determined state, extrinsically motivated, imposed, or marked by the thwarting of one or more psychological needs. By framing the experience of being alone through the lens of Self-Determination Theory, we highlight how the psychological processes that accompany it, an individual’s extrinsic versus intrinsic motivational stance, and need satisfaction or frustration, can explain why similar circumstances while being alone may be experienced as either enriching or distressing. Implications for psychological interventions, education, and research are discussed, suggesting that fostering autonomy-supportive environments and strengthening intrinsic motivation can promote beneficial solitude and reduce harmful loneliness. Understanding solitude and loneliness as outcomes of self-determined versus non-self-determined conditions offers a nuanced view of time spent alone, helping to differentiate between healthy and maladaptive forms and guiding strategies to enhance individuals’ psychological well-being.

## Introduction

Being with oneself and alone are often understood in objective terms, such as physical separation from others. Weinstein et al. call this neutral state „everyday solitude “ which is inherently not good or bad; they call it *“an empty vessel waiting to be filled”* or *“a mirror reflecting what we bring into it, what we experience there, and what we take away from it”* (Weinstein et al., 2024, pp. 186–187). The psychological experience of being with oneself has significant implications (both positive and negative) for mental health and well-being (Park et al., 2020). However, empirical and phenomenological evidence suggests that this condition is subject to internal interpretations that can transform the experience into one perceived as more or less pleasant, depending on individual psychological and contextual factors, such as beliefs, expectations, motivations, life circumstances, social context, or cultural norms (e.g., Rodriguez et al., 2025; Russell et al., 2012).

Within psychological research, it is therefore crucial to distinguish between more or less pleasant experiences, namely solitude and loneliness. Although often conflated, solitude and loneliness are conceptually and experientially distinct constructs with unique implications for psychological functioning. Their distinction has theoretical and clinical relevance, as the same physical state of being alone can induce markedly different psychological outcomes (Borg & Willoughby, 2022; Kumar, 2023; Rodriguez et al., 2025; Weinstein et al., 2021).

Although solitude lacks a universally accepted definition, most studies define it as a state of being alone that is often positively experienced as a desirable part of life, providing restorative and rewarding experiences (Long & Averill, 2003; Rodriguez et al., 2025; Weinstein et al., 2021). Similarly, solitude has been described as *“a state in which the self is placed intentionally at the center of one’s attention and, if not physically alone, then mentally distanced from others”* (Weinstein et al., 2024, p. 39). Importantly, solitude is often associated with numerous positive psychological outcomes, including enhanced creativity, self-reflection, personal growth, and well-being, as well as cognitive restoration and emotion regulation (Borg & Willoughby, 2022; Rodriguez et al., 2025; Zhou et al., 2023). Interestingly, spending less time alone than one would like, called aloneliness, is associated with increased stress and depression (Coplan et al., 2019).

Conversely, loneliness is a distressing subjective experience characterized by social disconnection, alienation from others, or unmet relational needs resulting from a perceived deficiency or discrepancy in the desired quantity or quality of one’s social relationships (Ernst et al., 2023; Hawkley & Cacioppo, 2010; Perlman & Peplau, 1981). These experiences can be transient (episodic) or prolonged and persistent over time, and they may be resistant to change (chronic; Lim et al., 2023). Loneliness is closely related, yet distinct from, social isolation, whereas loneliness is a subjective emotional experience, the latter is characterized as an objective and involuntary state of limited access to social support or networks (Hawkley & Cacioppo, 2010; Hong et al., 2023; McKenna-Plumley et al., 2023; Park et al., 2020). Loneliness has been extensively documented as a significant risk factor for diverse adverse outcomes accompanied by negative affective and cognitive patterns, including depression, anxiety, impaired sleep and cognition, cardiovascular health, poorer quality of life, and increased mortality risk, underscoring its clinical significance (e.g., Ernst et al., 2023; Holt-Lunstad et al., 2015; Park et al., 2020).

Recently, however, Nguyen and Rodriguez (2024) have warned that these two conflicting, pervasive narratives about being alone, namely loneliness and solitude, may oversimplify the experience and its multifaceted nature, as well as the complexity of its effects. Loneliness and solitude can be understood as extremes along a spectrum of experiences of being alone. Consequently, they propose a methodological framework that deconstructs the abstract concept of being alone into several key dimensions: frequency and duration, cognitive processes involved, types of activities performed, elicited emotional experiences, and underlying motivations.

In line with this proposed framework, we focus in this article on motivation as the most impactful and widely studied dimension influencing how individuals experience being alone. A broad range of motivational theories addressing solitude and loneliness emphasize the central role of motivational factors in determining whether time alone is experienced as enriching or aversive and help explain its outcomes. As reported by McCarthy et al. (2025), past and current research includes several theoretical foundations, motivational frameworks, and complementary models that highlight the human drive for connection and the motivational systems activated when this drive is thwarted. These include social exchange theories (Lawler, 2001), socio-psychological theories (e.g., cognitive discrepancy theory; Peplau & Perlman, 1982), and evolutionary theories (Cacioppo & Cacioppo, 2018), which have mostly focused on loneliness rather than solitude. The research has been further supported by other theoretical perspectives, such as the reaffiliation motive (RAM; Qualter et al., 2015), inclusion motivation (Kanterman et al., 2022), or the need to belong (Baumeister & Leary, 1995), all of which emphasize the role of unmet social needs in generating distress during time alone. Taken together, these perspectives affirm the centrality of motivational mechanisms in shaping responses to time alone and support the proposition that not all forms of being alone are equivalent, and that the underlying quality of motivation shapes the experience.

This insight aligns with the central ideas of an influential theoretical framework that offers particularly useful clinical and research insights, the Self-Determination Theory (SDT), with its emphasis on the basic psychological needs for autonomy, competence, and relatedness, and motivation that lie on a continuum of self-determination (Ryan & Deci, 2000b, 2017). SDT provides a coherent theoretical lens and validated methodological approach, as seen in instruments like the Relative Autonomy Index (e.g., Howard et al., 2020) and especially the Motivation for Solitude Scale (Nicol, 2006; Thomas & Azmitia, 2019), making this framework highly relevant for both clinical and research applications focused on loneliness and also solitude.

The present article aims to examine the experience of being alone from the SDT perspective, encompassing both solitude and loneliness. Specifically, the article summarizes the core tenets of SDT and elaborates on how its key ideas and theses are reflected both in the subjective experience of time spent alone and in the findings of contemporary studies. By examining these aspects, the article aims to elucidate how SDT can provide a coherent framework for understanding the experiences of being alone. Particular attention is given to the support and frustration of the three basic psychological needs (autonomy, competence, and relatedness), as well as other key SDT components, in understanding and shaping these experiences.

By distinguishing between self-determined and non-self-determined motivational processes, we seek to clarify the conditions under which being alone supports psychological well-being and is experienced as enriching, versus when it contributes to distress or disconnection. This conceptual analysis seeks to contribute to a more nuanced psychological model of being alone, with implications for both theory and applied efforts, such as within therapeutic and psychoeducational contexts, designed to reduce loneliness and foster adaptive solitude.

## Self-Determination Theory and Time Alone

Introduced over 40 years ago, the SDT has been a prominent theory of human motivation and personality development belonging to the broader family of Social Needs–Based Theories (e.g., McCarthy et al., 2025). SDT has been extensively applied across a range of domains, including healthcare, education, physical activity, digital media use, work, and organizational behavior. As a result, the theory rests on a well-established empirical foundation and an organismic framework with six formal „mini-theories “ and numerous side models that systematically overlap, and upon which novel additions and extensions continue to be developed (Ryan & Deci, 2019). Nonetheless, systematic research explicitly addressing time alone, solitude, and loneliness within the SDT framework has emerged only in recent years (e.g., Nguyen et al., 2022; Thomas & Azmitia, 2019; Weinstein et al., 2021).

As outlined by Ryan and Deci (2000b, 2019), SDT is a framework that emphasizes how social and environmental conditions can either support or hinder intrinsic motivation and internalization, while also highlighting the role of an individual's intrapersonal resources. At its core, SDT distinguishes between intrinsic motivation – engaging in an activity for its inherent satisfaction (e.g., spending time alone to reflect, create, or experience a sense of peace) – and extrinsic motivation, which refers to behavior driven by outcomes separable from the activity itself, such as rewards, external approval, status, or avoidance of guilt (e.g., being alone to escape social disapproval or anxiety). Self is considered an active integrative process that assimilates, coordinates, and regulates external (social and cultural) and internal (e.g., emotions, needs) inputs. Research on both intrinsic and autonomous motivation, as well as internalization processes, shows their enhancing effects on psychological integration, which supports healthy self-functioning, predicts positive experiences, and well-being outcomes (Ryan & Deci, 2019).

From the SDT perspective, intrinsic motivation is associated with and facilitated by the support of three innate and universal psychological needs: competence (the feeling of effectiveness and mastery), autonomy (the sense of volition and self-endorsement), and relatedness (the experience of social connection and belonging). When supported, these needs foster proactive engagement, persistence, and well-being, and in the context of our topic, promote solitude. Conversely, controlling contexts such as coercion, imposed rewards and punishments, guilt, or threats tend to suppress intrinsic motivation by undermining autonomy and other needs, contribute to various forms of psychopathology, and, consequently, for our topic, the time alone might be experienced negatively as loneliness (Ryan & Deci, 2000a, 2019; Weinstein et al., 2021).

Importantly, to further refine this intrinsic-extrinsic distinction, SDT identifies a continuum of motivational regulation that ranges from external regulation to fully self-determined forms of motivation (Howard et al., 2020; Ryan & Deci, 2000a). Within extrinsic motivation, the internalization continuum comprises four regulatory styles that we link in the present article to experiences of being alone. The first style is external regulation, in which behavior is experienced as controlled or alienated and motivated by external pressures, typically in the form of reward contingencies (e.g., monetary incentives) or punishments and coercion administered by others (e.g., being alone because of pressure from others; prison solitary confinement, or imposed isolation). Introjected regulation refers to behavior motivated by internalized controlling pressures, such as regulations, rewards, or punishments, in the form of ego-involved emotions, including pride, anxiety, shame, and guilt (e.g., spending time alone due to internalized guilt or shame). Identified regulation characterizes behavior in which individuals consciously accept the importance or value of an activity and engage in it willingly and volitionally, even when it is not inherently enjoyable (e.g., being alone for self-endorsed goals like reflection and other personally valued goals). Integrated regulation, the most autonomous form of extrinsic motivation, describes cases in which the behavior is assimilated into the self and experienced as congruent with one’s identity (e.g., time alone as an expression of one’s values and identity). In addition to these regulatory styles, amotivation represents a state characterized by a lack of intention, perceived competence, or sense of value in one’s behavior (e.g., staying alone being perceived as a meaningless activity). Finally, at the end of the continuum lies intrinsic motivation, in which behavior is pursued solely for the inherent enjoyment and interest it provides, such as voluntarily engaging in creative solitude for personal enjoyment or growth. This motivational continuum enables us to differentiate not merely between whether people are motivated to be alone, but also why, which influences the psychological outcomes.

**Fig. 1:**
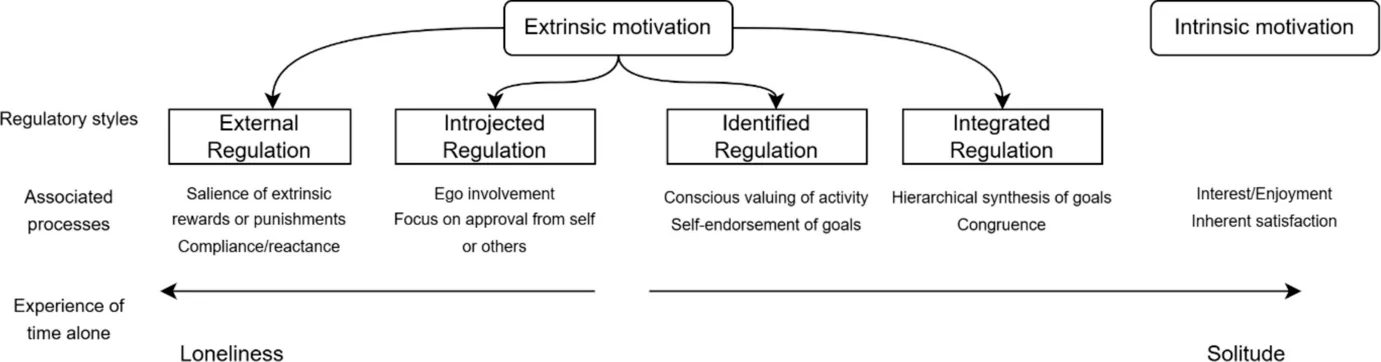
Continuum of motivational regulation and corresponding experience of time alone

This continuum of motivational regulation is shown in Fig. 1 (adapted from Ryan & Deci, 2000a). We argue that the perception of time spent alone can also be situated along this continuum, with the qualitative shift between loneliness and solitude becoming particularly salient at the transition from the introjected to identified regulation. Within this framework, time alone that is predominantly externally regulated is more likely to be experienced negatively and to correspond with feelings of loneliness. Conversely, the time alone that is increasingly intrinsically motivated is more likely to be experienced positively as solitude. This is also supported by previous studies demonstrating that non-self-determined experiences of being alone are associated with loneliness and other adverse psychological outcomes (e.g., Zyl et al., 2018).

To illustrate the complexity of the topic, it is important to note that individuals’ inner capacities and intrapersonal characteristics also play a role in transforming, framing, and interpreting objective circumstances as either positive or negative experiences (e.g., a prisoner who is externally regulated and placed in solitary confinement may nonetheless be capable of subjectively transforming this situation into an experience of positive solitude). Taken together, SDT and its interrelated mini-theories and models that have been developed over time (e.g., Ryan & Deci, 2019) clarify how the nature and quality of motivation shape human experiences, including experiences of being alone. Central to SDT is the principle that motivation does not arise in a vacuum; rather, intrinsic and autonomous motivation is nurtured or undermined by the extent to which the basic psychological needs for autonomy, competence, and relatedness are satisfied. Extensive SDT research shows that these needs function as essential nutrients for psychological growth and well-being, and that their satisfaction also determines whether time spent alone is experienced as restorative, liberating, and self-determined, or as isolating, painful, and distressing. In the next section, these needs are examined in detail to illustrate how their satisfaction or frustration shapes the subjective experience of being alone.

## Autonomy, Competence, Relatedness, and Time Alone

From the SDT perspective, autonomy, competence, and relatedness are essential for motivation and healthy self-functioning (Ryan & Deci, 2000a, 2019). Support for all three needs facilitates the internalization and integration of behavioral regulation, allowing people to feel competent and connected, experience more positive outcomes, and engage in activities with greater self-determination and autonomous motivation (Ryan & Deci, 2000a, 2019). When individuals feel that they can act in a volitional and self-congruent way (autonomy), experience themselves as capable and effective in pursuing personally meaningful goals (competence), and feel connected to and valued by others (relatedness), they tend to thrive across a wide range of well-being indicators (Adams & Weinstein, 2024).

In the context of solitude and loneliness, these psychological needs take on particular importance. In the following paragraphs, the satisfaction and frustration of each need are examined in relation to both solitude and loneliness, guided by the following questions: In what ways do autonomy, competence, and relatedness shape the subjective experience of time spent alone? How and under what conditions can each need be satisfied or frustrated, and what are the implications for the experience?

### Autonomy

SDT defines autonomy as the sense of volition and self-congruence in one’s actions – that is, feeling and experiencing that one’s behavior originates from oneself rather than from external or internalized pressures and demands (Ryan & Deci, 2000a; Weinstein et al., 2023). Voluntary withdrawal into solitude, therefore, represents an expression of autonomy that distinguishes beneficial solitude from enforced isolation or involuntary loneliness. Research by Weinstein et al., (2023, 2024) has identified three autonomy-related characteristics of solitude: perceived choice and volition (being free to do what one wants, supporting self-structure), freedom from pressure (absence of external or internal demands, judgments, and expectations), and self-connection and authenticity (being free to be oneself, supporting quietude, increasing closeness and intimacy with oneself, leading to greater self-integration and self-understanding). Since time alone usually reduces immediate social demands and distractions, it can create opportunities and conditions for self-reflection and connection that satisfy the need for autonomy and support personal growth within the SDT tradition. In such contexts, people can decide how to structure their time, pursue personally meaningful activities and goals, or simply pause and reflect, which enhances their sense of agency and intrinsic motivation (e.g., Ryan & Deci, 2019; Sheldon et al., 2004).

Solitude can thus become a setting in which autonomy is actively fostered through a perceived choice, freedom from pressure, social conformity, and external judgment. This creates opportunities for authentic self-engagement, feeling a sense of closeness and intimacy with oneself, and gaining clearer insight into personal values, priorities, and desires (Weinstein et al., 2023). Borg and Willoughby (2022) and Rodriguez et al. (2025) demonstrated that individuals with a high affinity for solitude who perceived their time alone as self-chosen and personally valued reported fewer depressive symptoms and lower loneliness, as well as better friendship quality, and higher self-esteem. This suggests that spending time alone may be advantageous for gaining insight into one’s thoughts and feelings and for engaging in personally meaningful independent pursuits. Autonomously motivated solitude – time alone that is freely chosen and appreciated for its personal benefits – has also been linked to greater enjoyment, self-awareness, and self-esteem, as well as energizing and revitalizing effects. Moreover, the mere opportunity to choose how to live moment-to-moment was perceived as rewarding (Coplan et al., 2019; Moller et al., 2006; Nguyen et al., 2019; Weinstein et al., 2023). Similarly, studies indicate that individuals with a stronger dispositional tendency toward autonomy derive greater benefits from daily solitude, even if they generally do not prefer being alone (Weinstein et al., 2023).

Conversely, frustration of the need for autonomy, when time alone is imposed or entered into under controlling conditions, can transform solitude into a potentially aversive state akin to loneliness. Individuals who withdraw because of external expectations (e.g., social stigma, family demands, social exclusion) or internalized pressures (e.g., guilt, shame, avoidance of feared evaluation) experience being alone with an externally perceived locus of causality (DeCharms, 1968), thereby undermining intrinsic motivation and self-connection (Ryan & Deci, 2000a; Weinstein et al., 2023). Weinstein et al. (2023) also argue that, because time alone is relatively free of sensory distractions, social demands, and structure, it may be particularly challenging for individuals who are self-alienated, defensive, or low in autonomous orientation. Under such conditions, being alone may be experienced as lonely, empty, boring, or even alienating rather than restorative.

Supporting this, Zhou et al. (2023) identified several solitude profiles among adolescents, with 38% of their sample (N = 355) characterized by negative motivational solitude, marked by the highest aversion to being alone and the lowest level of autonomous motivation. These individuals disliked being alone yet felt forced to remain alone for extrinsic reasons. They also showed psychological maladjustment (i.e., high levels of loneliness, depressive symptoms, and low levels of basic needs satisfaction). Moreover, they tended not to accept their current state of being alone, consider solitude worthless, and doubted their capacity to be alone. These distinctions help explain why two people can be in the same physical state of “being alone” yet experience it in opposite ways – one as autonomy-supportive solitude, the other as autonomy-frustrating loneliness. This underscores the SDT’s emphasis on the quality of motivation and the importance of fostering autonomy-supportive conditions and volitional choice to promote the beneficial aspects of solitude and reduce the risk of loneliness.

### Competence

The second need, competence, refers to the feeling of mastery and the sense that one can succeed and grow (Ryan & Deci, 2020). Competence is another pivotal factor that can shape whether time spent alone is experienced positively or negatively. In the context of solitude, this need is often satisfied through engagement in personally meaningful activities that affirm skills and mastery, particularly within well-structured environments that provide optimal challenges, positive feedback, and opportunities for growth (Ryan & Deci, 2020). Together with the absence of demeaning evaluations, such conditions are predicted to facilitate internalization and intrinsic motivation. For example, positive feedback enhances a feeling of competence, which in turn supports intrinsic motivation (Ryan & Deci, 2000a, 2019). Consequently, time spent alone is more likely to be experienced positively as solitude.

Weinstein et al (2021) reported that competence satisfaction during time spent alone was associated with greater felt efficacy, a focus on skill development, and engagement in (daily) activities. When individuals choose to spend time alone and structure their days, they can use this opportunity to pursue hobbies, creative projects, or intellectual endeavors that require concentration and effort, thereby fostering a sense of accomplishment and self-efficacy. Such competence-building activities may include learning, reading, cooking, playing, completing homework, exercising, hiking, writing, painting, problem-solving, or engaging in mindfulness practices, all of which contribute to positive self-regard and psychological resilience (Weinstein et al., 2021, 2023; Zhou et al., 2023). These endeavors not only sustain competence satisfaction but also enrich the subjective experience of being alone by infusing it with purpose and reinforcing a sense of personal agency and capability. Borg and Willoughby (2022) argue that self-reflection and creative pursuits represent positive motivations for spending time alone. Experiencing quietude and being in a quiet space, both of which are connected to the solitude experience, may be particularly important for accessing one’s thoughts, making volitional choices, expressing interests and skills, and learning about oneself (Ryan & Deci, 2000a).

The above-mentioned findings suggest that time spent alone can be transformed into solitude by focusing on the satisfaction of competence. Competence in this context refers not only to task-related mastery but also to feeling capable of being alone with oneself, as elaborated by Weinstein et al. (2023). This sense of competence is related to emotional and cognitive self-regulatory capacities, such as understanding one’s own preferences, interests, thoughts, salient emotions, and values, as well as the ability to create structure and set achievable, self-congruent goals aligned with personal values. Weinstein et al. (2023) further argue that activities that provide a sense of pace or progress, along with feedback, even simple activities like preparing meals, reading, or maintaining daily routines, or regularly calling/messaging a relative or friend, can transform time alone into a positive experience of solitude. Such activities can provide something to look forward to and facilitate rewarding experiences while alone. This competence in self-management may not only enhance current well-being but also equip individuals with skills to cope more effectively with future stressors, strengthen persistence and creativity, and promote deeper learning. These processes support self-understanding, emotional clarity, and personal integration, thereby promoting personal growth, increasing satisfaction, and reinforcing the adaptive value of self-determined solitude.

By contrast, from the intrapersonal perspective, individuals who are unprepared to be alone or unable to create structure and direction when alone (e.g., by pursuing too many goals mindlessly or compulsively) may experience frustration of competence need. Such frustration may manifest as feelings of self- and other-alienation, boredom, or anxiety, and increase the likelihood that being alone is experienced as loneliness. In these circumstances, being alone does not support self-congruence and internal coherence; instead, it highlights a perceived lack of competence in self-connection or in relating to others (Weinstein et al., 2023).

Competence frustration is also connected to the interpersonal perspective of solitude and loneliness. As studies show, difficulties in navigating social contexts or seeking support, often rooted in deficits in social skills or perceived social inadequacy, can thwart competence needs and contribute to loneliness. Individuals who perceive themselves as socially incompetent, lack confidence in interacting with others, experience peer difficulties, report low self-esteem, feel they have little to offer in relationships, distrust others, or fear negative evaluation may be more likely to avoid social interactions. Such avoidance further limits opportunities to develop and sustain relational competence, creating a barrier to reducing loneliness (Borg & Willoughby, 2022; Frerichs et al., 2020). Over time, this pattern can result in a self-fulfilling cycle in which limited social experience impairs skill development, thereby reinforcing feelings of incompetence, passivity, disengagement, isolation, and loneliness (Hawkley & Cacioppo, 2010; Weinstein et al., 2023).

### Relatedness

Whereas autonomy and competence primarily concern one’s capacity for self-direction and growth, relatedness focuses on the fundamental human need for belonging and connection, including the desire to be cared for, respected, and significant to others (Ryan & Deci, 2020). Relatedness may also manifest intrapersonally, in one’s relationship with oneself, maintaining a sense of internal relatedness or peace in one’s own company. This need is not fulfilled solely through immediate social contact but may also be satisfied through a broader sense of connection.

Given that being alone is, by definition, characterized by the physical absence of others, the role of relatedness in this context is natural and highly relevant. Relatedness represents a bridge between the self and the social world, shaping how individuals experience and interpret both being alone and social interaction. It influences not only social engagement but also internal motivation, shaping how individuals interpret and respond to social contexts, including time spent alone. As emphasized by Ryan and Deci (2000a), extrinsically motivated behaviors often derive their meaning and persistence from the sense of connectedness, in which people are willing to pursue certain goals because they are valued by others or serve to maintain belonging within meaningful relationships (e.g., family, peers, or broader social and cultural groups). A sense of relatedness and belonging thus provides a foundation for psychological integration and internalization. When individuals feel securely attached to and accepted by others, they are more likely to adopt values and behaviors that are socially endorsed.

Therefore, we argue that relatedness may support not only social bonds but also the inner capacity to experience time alone without feeling lonely. In this way, time alone can become less extrinsically and increasingly intrinsically motivated, moving along the internalization continuum described earlier (see Fig. 1). In other words, when individuals initially spend time alone for extrinsic reasons and experience loneliness, supportive social contexts and experiences of relatedness (such as appreciation, modeling, and discovering its positives) may facilitate the gradual internalization of time alone and recognition of its benefits, transforming loneliness into solitude.

In the context of relatedness, Weinstein et al. (2023) argue that time alone allows individuals to step outside the “social dance” of daily interactions, in which expectations, comparisons, and judgments, along with external and internalized pressure, shape behavior, thoughts, and emotions. In their research, primary motives for spending time alone related to relatedness included the absence of social noise, freedom from external and introjected pressures, self-connection, and authenticity. When time alone is experienced as solitude, relatedness may support independence from others, reduce social fatigue, help recharge the so-called “social battery” and restore balance. Thus, solitude offers a pleasant freedom from social dynamics, demands, and introjected self-judgments. Consequently, individuals can engage in self-reflection, reconnect with personal emotions, values, and desires, experience feelings of connection to the world, joy, wonder, and awe, make personally meaningful choices about what to do and how to act, and pursue activities that interest them (Weinstein et al., 2023, 2024, pp. 65–86). This closely relates to the need for autonomy discussed earlier. In addition, deliberate withdrawal as part of the cyclical process of social withdrawal and reconnection with others can facilitate perspective-taking and self-other differentiation, thereby enhancing interpersonal sensitivity and empathy upon returning to interactions with others (e.g., Simion, 2025; Weinstein et al., 2024, pp. 110–111).

This inward turn does not negate the significance of others; rather, it represents a complementary mode of connection grounded in self-awareness, authentic self-connection, and emotional attunement. Meaningful solitude enables individuals to reflect and listen to their inner “voices” without the echo of external evaluation, offering a restorative and relational experience of me- or solo- time (e.g., Ost Mor et al., 2021; Palgi et al., 2021; Shiovitz-Ezra & Rozen, 2024). We hypothesize that these inner experiences may include memories of positive interactions, securely internalized representations of caring others, or reflections on significant role models perceived as autonomy-supportive (e.g., parents, teachers, significant others). Individuals may engage in inner dialogues with these figures, whether living, deceased, historical, fictional (including those from films, literature, games, etc.) or religious and divine (through practices such as prayer), to further internalize and deepen their connection to their guiding values, principles, religion, beliefs, or philosophies, and promote better self-understanding.

High-quality dyadic relationships in adulthood are characterized by autonomy, also expressed as voluntary mutuality (Ryan & Deci, 2019). Experiences of such support and voluntariness can promote greater interpersonal openness, sharing, and use of social support, all of which are associated with enhanced psychological well-being (e.g., Roth et al., 2019). In this context, Weinstein et al., (2024, p. 191) highlight the role of social self-efficacy, noting that people can carry the benefits of good relationships into their alone time.

Naturally, frustration, unfulfillment, or absence of the above-mentioned needs and factors have distinct implications for how time spent alone is perceived. Under such conditions, being alone may become a context in which disconnection, a sense of not belonging, and the absence of care or significance are amplified, leading to feelings of loneliness. Studies by Weinstein et al., (2023, 2024) provide several insights into how this process may unfold. One contributing factor may be social exclusion, i.e., ostracism by others, rejection, or stigmatization. Experimental studies have shown that individuals who experienced rejection prefer spending time alone rather than with those who rejected them (e.g., Ren & Evans, 2021; Zhang & Hackel, 2026). However, such time alone is typically not self-endorsed; it reflects external or introjected regulation when considered within SDT’s internalization continuum described earlier. Similarly, when processes relevant to relatedness are disrupted, absent, or inverted, potential benefits of solitude are undermined, and feelings of loneliness are more likely to emerge. Specifically, the „social dance “ of engagement may become distorted when people constantly adjust themselves to meet external expectations, lose sight of themselves and their own needs, emotions, or desires. When such individuals are alone, they may experience a profound absence of direction or identity, highlighting an unmet desire for genuine connection with others and with themselves (Weinstein et al., 2024, p. 122).

Additionally, deviations in the intensity and frequency of social engagement can lead to mutual alienation and loneliness. For example, when individuals are unable to withdraw from social interactions and adequately recharge, it may produce social fatigue and intrusive social noise, contributing to loneliness and aloneliness. In contrast, when social encounters are too infrequent, it can create withdrawal that disturbs reconnection. Moreover, one’s environment, other people, and close ones can exert external pressure, impose unrealistic expectations, and make harsh comparisons and critical judgments, as shown in previous research (e.g., Heissler et al., 2025; Weinstein et al., 2024, p. 60). Consequently, time spent alone may no longer be a source of relief or self-connection. Instead, it may become an unwanted experience that individuals actively seek to avoid, as it can be associated with impaired self-perception, negative thoughts, ruminations, and inner experience dominated by guilt, self-accusations, internalized demands, and pressures rooted in past relationships.

Arguably, such experiences may also be linked to the absence of autonomy-supportive figures, role models, or caring people who might facilitate inner restorative and relational experiences during time alone, especially during critical developmental periods. The lack of such encounters, together with unfulfilled longing for connection, may negatively influence the quality of me- or solo-time. In fact, these relational dynamics are strongly reflected in widely used loneliness measures, such as not feeling close to anyone, being misunderstood, withdrawn, rejected, excluded, or lacking companionship (e.g., UCLA Loneliness Scale; Russell, 1996; DeJong Gierveld Loneliness Scale; de Jong-Gierveld & Kamphuls, 1985).

### Summary

To summarize this section, the SDT needs for autonomy, competence, and relatedness help explain why time spent alone can be experienced either as solitude or as loneliness, depending on the satisfaction or frustration of these interrelated basic psychological needs. Autonomy shapes whether being alone is experienced as volitional and self-congruent or as imposed and constraining, i.e., when individuals feel they have chosen it. Competence influences individuals’ capacity to structure, direct, and meaningfully engage with their time alone and manage the feelings it evokes. Finally, relatedness remains important even in the absence of others, as internalized relational experiences and the sense of (para)social connection strongly color the subjective quality of being alone.

When these needs are satisfied, time alone can foster restoration, self-integration, and enhance well-being; when they are frustrated, being alone is more likely to be experienced as loneliness, with all its negative consequences. Thus, loneliness does not arise merely from physical aloneness but from unmet psychological needs within the context of being alone. Understanding these need-based dynamics provides a nuanced way to think about both the risks and the potential of being alone.

## Implications and Limitations

The SDT-based perspective on solitude and loneliness presented in this article offers a reconsideration of how these are conceptualized across research, practice, and everyday discourse. Rather than viewing time spent alone as inherently maladaptive or uniformly beneficial, this perspective highlights the conditions under which time alone supports psychological well-being versus when it becomes distressing, through the satisfaction or frustration of autonomy, competence, and relatedness. Thus, adopting the SDT perspective enables a meaningful reframing of being alone by shifting away from deficit-based and pathologizing interpretations toward motivational and need-based understanding. This reframing has important implications for reducing stigma and strengthening prevention efforts. It challenges the rooted assumptions that being alone is uniformly harmful, allowing individuals to approach time alone with greater openness and intentionality.

However, this shift in perspective is not unprecedented, and we may once again find ourselves in a period in which being alone is not perceived only negatively, with modern research and theory providing more refined tools for its description and understanding. As described by Weinstein et al., (2024, pp. 13–33), historical and cultural views of being alone have long been ambivalent, reflecting mankind’s complicated relationship with it. Across time, being alone has been idealized and reserved for the powerful or privileged, such as philosophers, poets, or spiritual and religious figures (e.g., prophets, hermits, monks), and also feared as empty, shameful, undesirable, evolutionarily dangerous for our species, bad for health, and pathological. Thus, being alone has been both worshipped and feared, discouraged and tolerated, romantically desired or actively avoided, and framed as both exceptional and extreme in good and bad ways. SDT allows solitude to be understood as a potentially ordinary and adaptive aspect of everyday life. It also helps find balance between solitary and communal conditions, being and doing, and ultimately fostering human flourishing (Weinstein et al., 2024, p. 217).

The following section thus outlines the broader implications of this approach while also critically considering the boundaries and limitations of SDT as an explanatory framework for understanding solitude and loneliness. As a guiding framework, we will focus on the SDT continuum of motivation in Fig. 1, from external and introjected regulation through identified and integrated regulation to intrinsic motivation. With the qualitative shift between loneliness and solitude appearing at the transition from the introjected to identified regulation, this perspective can have several implications. Taking into account also the described basic psychological needs, they offer a useful lens for understanding qualitative differences in how time alone is experienced.

Across research, education, and psychological care, it is important to identify and understand an individual’s current motivational stance toward time alone and their implicit beliefs and attitudes. This approach should also consider how this stance interacts with the satisfaction or frustration of autonomy, competence, and relatedness needs, rather than treating loneliness or solitude as fixed states. Once loneliness and solitude are understood as emerging from different motivational regulations of time alone and needs, it becomes possible to move beyond a predominant focus on loneliness (and social isolation), allowing greater attention to solitude as a distinct psychological experience. Moreover, both phenomena should be examined across the lifespan from childhood to older adulthood, including the identification of different solitude and loneliness profiles and their motivational underpinnings (e.g., Zhou et al., 2023). Such a shift in focus may also revise or temper often misleading media narratives about the so-called “loneliness epidemic” (Ortiz-Ospina & Roser, 2019). This approach also underscores the importance of incorporating measures that assess not only relatedness and the quantity and quality of social contact and networks, but also autonomy, competence, and the qualitative experience of time alone. These dimensions can be captured by instruments assessing motivational processes, such as the Motivation for Solitude Scale (MSS; Nicol, 2006) and its shortened version, MSS-Short Form (Thomas & Azmitia, 2019). However, in their current form they are primarily focused on assessing the degree of self-determination, which highlights the need for the development of new methods and approaches capable of capturing the motivational processes. One promising avenue may be to infer an individual’s position on the extrinsic-intrinsic motivation continuum by triangulating findings and extrapolating from existing data sources, including self-report measures, interviews, or behavioral observations. Then cross-reference them with the defining characteristics and markers of extrinsic and intrinsic motivation types, in order to approximate where an individual falls along this motivational continuum. Together, such approaches acknowledge that the psychological impact of being alone depends less on its frequency or duration and more on how time alone is motivated and subjectively experienced.

This framework is applicable across educational, psychoeducational, and therapeutic contexts, supporting gradual shifts along the motivational continuum toward more self-determined and intrinsically motivated solitude, for example through deliberate attention to the described SDT’s psychological needs. This was demonstrated by previous studies applying SDT principles across diverse settings and domains (e.g., Manninen et al., 2022; Ryan & Deci, 2019; Wang et al., 2024). Educational and therapeutic settings can naturally converge in their emphasis on normalizing and reframing being alone as a learnable skill and developmental capacity rather than a deficit or risk state. On this basis, interventions can support more personalized and targeted mental health care and aim at cultivating healthier and more self-determined relationships with time alone. Rather than assuming that reduced aloneness is always the desired outcome, educators, clinicians, and therapists can focus on strengthening autonomy, competence, and both interpersonal and intrapersonal relatedness, thereby alleviating non-self-determined forms of being alone. This includes addressing maladaptive beliefs about time alone, using cognitive reappraisal, identifying and reducing external demands and self-imposed pressures, some of which show to be in line with already existing effective interventions reducing loneliness (Masi et al., 2011). Additionally, fostering reflective understanding of personal needs, autonomy-supportive coping strategies and narratives, and strengthening competencies for structuring time alone so that it becomes increasingly self-endorsed rather than imposed or avoidant. These tailored approaches respect individual differences in affinity for being alone and psychological readiness to engage with solitude in a more self-determined manner. As discussed in the section on relatedness, the positive influence of others may support the intrinsic motivation to spend time alone and discover its potential benefits. Emerging evidence suggests that interventions targeting individuals’ mindsets about solitude, specifically how they think about being alone, the expectations they hold, and their perceived capacity to engage with “me” time, can reduce loneliness and improve mental health outcomes (Rodriguez et al., 2025; Weinstein et al., 2024, pp. 107–108, 190). Importantly, such approaches emphasize engaging in intrinsically enjoyable or personally meaningful activities during time alone, rather than focusing exclusively on increasing social interactions. Research also identified factors that promote resilience in aloneness, including optimism, a growth-mindset, self-compassion, curiosity, and present-moment awareness (Weinstein et al., 2024, p. 192), pointing to concrete intervention targets. Framing solitude as a potential opportunity for restoration, autonomy, self-connection, or personal growth may help normalize time spent alone and reduce associated stigma.

However, interventions must remain sensitive to developmental stages and cultural contexts, as the meanings and functions of solitude, as well as the salience of psychological needs, shift across the lifespan. For example, among children and younger adults, solitude may provide space for enjoyable activities, planning, emotion regulation, temporary social disengagement, and regaining a sense of control. In contrast, older adults often emphasize the quality of their relationships and the freedom to choose when and how to be alone, showing the importance of autonomy and authenticity. Across age groups, time alone may also foster peacefulness by engaging with nature, hobbies, or recreational activities that support creativity and self-expression (Weinstein, 2024, pp. 212–213).

Finally, these considerations extend beyond formal interventions into everyday life and relationships. As we recognized the important role of significant others, role models, and autonomy-supportive figures, such as parents, partners, teachers, coaches, peers, and even parasocial relationships, we highlight the broader social responsibility to create these autonomy-supportive environments. By modeling and supporting healthy engagement with both connection and solitude, individuals can contribute not only to their own well-being but also to that of others, fostering cultural narratives that value positive alone time without equating it with withdrawal or loneliness. In other words, the SDT highlights the importance of both having and being an autonomy-supportive figure in one’s social environment.

Despite its substantial explanatory power, SDT has several known boundaries and limitations as a framework. As noted by, e.g., Gupta and Behl (2025), criticisms focusing on SDT’s emphasis on autonomy and basic psychological needs, especially with respect to cultural variability and differences between individualistic and collectivistic cultures, are recurrent. However, as demonstrated by Ryan and Deci (2020), a substantial body of empirical evidence challenges and, in many cases, refutes these claims, and proving those needs as universal. From a methodological standpoint, difficulties in operationalizing SDT’s motivational continuum have been highlighted (e.g., Chemolli & Gagné, 2014). Distinguishing among intrinsic, integrated, identified, introjected, and external forms of regulation is often problematic and underscores the need for the development of valid theory-driven measures and corresponding scoring systems. Gupta and Behl (2025) also mention that the theory may also underestimate the potential value of well-designed extrinsic incentives, particularly in tasks that are inherently low in interest, and excessive focus on autonomy can lead to decision fatigue and reduced performance. Additionally, they note that SDT remains relatively under-integrated with broader psychological frameworks such as the Big Five, limiting its interdisciplinary insights. This might also raise questions about the theory’s conceptual alignment with other prominent approaches to loneliness, solitude, and time alone, including social exchange theories, socio-psychological theories, or evolutionary perspectives (McCarthy, 2025). It may also underrepresent broader or specific structural, socioeconomic, and cultural constraints and mechanisms that shape opportunities for autonomy, competence, and relatedness building, including factors such as poverty, discrimination, disability, caregiving burden, and institutional living arrangements, which contribute to the aforementioned continuum of motivation. Finally, McAnally and Hagger (2024) mention the lack of formal testing of the mechanisms underlying SDT-based interventions. This raises questions about whether behavioral and subsequent outcomes can be attributed to the proposed theoretical processes, highlighting the need for intervention research to explicitly test mediation effects.

## Conclusion

Being alone is a subjective experience, profoundly affected by the motivations that guide it, underscoring that not all forms of time alone are equivalent. Through the lens of Self-Determination Theory, this article has sought to untangle these experiences, revealing how the related factors and satisfaction and frustration of basic psychological needs for autonomy, competence, and relatedness shape an individual’s relationship with time alone. By distinguishing between self-determined and non-self-determined motivations, SDT offers a solid theoretical foundation for understanding when and why time spent alone can foster growth, creativity, and well-being, or conversely, lead to feelings of loneliness and distress. Our analysis highlights that solitude, when chosen and aligned with intrinsic motivations, can serve as a context for self-reflection, personal growth, and the restoration of psychological resources. In such cases, being alone is not merely the absence of others, but an active, meaningful engagement with the self, supported by a sense of autonomy, competence, and both intra- and inter-relatedness. Conversely, loneliness often arises when time alone has extrinsic motivation, is imposed rather than chosen, when the individual feels incompetent in managing this experience, or when the need for relatedness is thwarted, undermining well-being and exacerbating feelings of disconnection. Contemporary research underscores these dynamics, demonstrating that the quality of being alone is less about physical separation and more about the psychological processes that accompany it.

The implications of this perspective extend beyond academic inquiry. For clinicians, educators, therapists, and practitioners, recognizing the role of intrinsic motivation and basic psychological needs in shaping experiences of time alone can help to tailor and cultivate self-determined motivations for spending time alone. By fostering environments that support intrinsic motivation through satisfying autonomy, competence, and relatedness, and building skills to satisfy needs even in the absence of physical company, it is possible to mitigate loneliness and harness the potential benefits of solitude.

Future research should address SDT’s limitations, especially by refining measures to better capture the motivational continuum, developing strategies to help individuals navigate time alone in ways that promote well-being and resilience, and rigorously testing SDT-based intervention mechanisms to clarify its applicability and enhance interdisciplinary insights. All to further refine SDT’s practical and theoretical contributions to the study of solitude and loneliness.

In sum, this article advocates for a more nuanced model of being alone, one that recognizes the diversity of experiences and the pivotal role of motivation. Pursuing the outlined directions could not only deepen our theoretical grasp of solitude and loneliness but could also translate into more effective, developmentally and culturally appropriate targeted interventions. Ultimately, such advances hold the promise of normalizing time spent alone, reducing associated stigma, and helping people transform being alone, whether chosen or circumstance-driven, into an opportunity for resilience, meaningful connection, and personal flourishing.

## Data Availability

No datasets were generated or analysed during the current study.
